# Comprehensive metabolomic and epigenomic characterization of microsatellite stable *BRAF-*mutated colorectal cancer

**DOI:** 10.1038/s41388-025-03326-y

**Published:** 2025-03-18

**Authors:** Aurora Taira, Mervi Aavikko, Riku Katainen, Eevi Kaasinen, Niko Välimäki, Janne Ravantti, Ari Ristimäki, Toni T. Seppälä, Laura Renkonen-Sinisalo, Anna Lepistö, Kyösti Tahkola, Anne Mattila, Selja Koskensalo, Jukka-Pekka Mecklin, Jan Böhm, Jesper Bertram Bramsen, Claus Lindbjerg Andersen, Kimmo Palin, Kristiina Rajamäki, Lauri A. Aaltonen, iCAN, Aurora Taira, Riku Katainen, Niko Välimäki, Ari Ristimäki, Toni T. Seppälä, Laura Renkonen-Sinisalo, Anna Lepistö, Selja Koskensalo, Kimmo Palin, Kristiina Rajamäki, Lauri A. Aaltonen

**Affiliations:** 1 Medicum/Department of Medical and Clinical Genetics, University of Helsinki, Helsinki, 00014 Finland; 2 Applied Tumor Genomics Research Program, Research Programs Unit, University of Helsinki, Helsinki, 00014 Finland; 3 Institute for Molecular Medicine Finland (FIMM), HiLIFE, University of Helsinki, Helsinki, Finland; 4 Molecular and Integrative Biosciences Research Programme, Faculty of Biological and Environmental Sciences, University of Helsinki, FI‐00014 Helsinki, Finland; 5 Department of Pathology, HUSLAB, HUS Diagnostic Center, University of Helsinki and Helsinki University Hospital, Helsinki, 00014 Finland; 6 Department of Surgery, Helsinki University Central Hospital, Hospital District of Helsinki and Uusimaa, Helsinki, 00290 Finland; 7 Department of Gastroenterology and Alimentary Tract Surgery, Tampere University Hospital and TAYS Cancer Centre, 33520 Tampere, Finland; 8 Faculty of Medicine and Health Technology, Tampere University, Tampere, 33100 Finland; 9 iCAN Digital Precision Cancer Medicine Flagship, University of Helsinki, Helsinki, 00014 Finland; 10 Department of Surgery, The Wellbeing Services of Central Finland, Hoitajatie 1, 40620 Jyväskylä, Finland; 11 The HUCH Gastrointestinal Clinic, Helsinki University Central Hospital, Helsinki, 00280 Finland; 12 Department of Education and Research, The Wellbeing Services of Central Finland, Hoitajatie 1, 40620 Jyväskylä, Finland; 13 Department of Sport and Health Sciences, University of Jyväskylä, 40014 Jyväskylä, Finland; 14 Department of Pathology, The Wellbeing Services of Central Finland, Hoitajatie 1, 40620 Jyväskylä, Finland; 15 Department of Molecular Medicine, Aarhus University Hospital, DK-8200 Aarhus, Denmark; 16 Department of Clinical Medicine, Aarhus University, DK-8200 Aarhus, Denmark

**Keywords:** Cancer genetics, DNA methylation, Mutation, Cancer metabolism

## Abstract

Oncogenic codon V600E mutations of the *BRAF* gene affect 10–15% of colorectal cancers, resulting in activation of the MAPK/ERK signaling pathway and increased cell proliferation and survival. *BRAF-*mutated colorectal tumors are often microsatellite unstable and characterized by high DNA methylation levels. However, the mechanistic link between *BRAF* mutations and hypermethylation remains controversial. Understanding this link, particularly in microsatellite stable tumors is of great interest as these often show poor survival. We characterized the metabolomic, epigenetic and transcriptomic patterns of altogether 39 microsatellite stable *BRAF-*mutated colorectal cancers. Metabolomic analysis of tumor tissue showed low levels of vitamin C and its metabolites in *BRAF-*mutated tumors. Gene expression analysis indicated dysregulation of vitamin C antioxidant activity in these lesions. As vitamin C is an important cofactor for the activity of TET DNA demethylase enzymes, low vitamin C levels could directly contribute to the high methylation levels in these tumors by decreasing enzymatic TET activity. Vitamin C transporter gene *SLC23A1* expression, as well as vitamin C metabolite levels, were inversely correlated with DNA methylation levels. This work proposes a new mechanistic link between *BRAF* mutations and hypermethylation, inspiring further work on the role of vitamin C in the genesis of *BRAF-*mutated colorectal cancer.

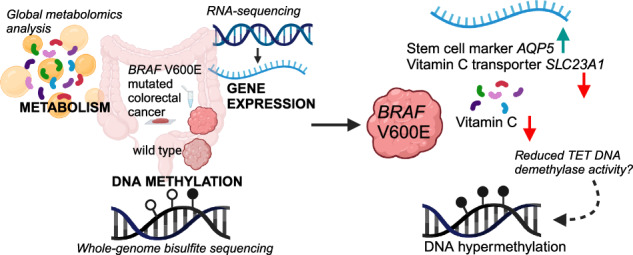

## Introduction

Somatic mutations in the *BRAF* gene occur in a variety of human cancers including melanoma and colorectal cancer (CRC) [1]. The vast majority of *BRAF* mutations cause a single amino acid change at codon 600 (V600E), constitutively activating the RAS-RAF-MEK-ERK-MAP signaling pathway. Overactivation promotes tumorigenesis by for example increasing cell proliferation and suppressing apoptosis [2].

*BRAF* mutations affect ~10-15% of sporadic CRCs. *BRAF*-mutated CRCs often belong to consensus molecular subtype 1 (CMS1), which is associated with both CpG island methylator phenotype (CIMP) and microsatellite instability (MSI) [3]. CIMP may be less common in microsatellite stable (MSS) compared to MSI *BRAF*-mutated tumors [4] yet some reports show only subtle differences [5], perhaps owing to differences in patient cohorts and CIMP panel markers utilized. CIMP and MSI phenomena are however often linked, as most CIMP CRCs are characterized by promoter CpG island methylation of the mismatch repair gene, *MLH1*, resulting in its transcriptional inactivation and MSI [6]. The prevalence of *BRAF* mutations is much higher among MSI vs. MSS tumors (~52–54% vs. ~5%, respectively) [5, 7]. MSS and MSI *BRAF*-mutated tumors comprise ~4.5% and ~4.9%-7.9%, respectively, of all sporadic CRCs [5, 7]. Importantly, MSI tumors show good survival independent of V600E status, while *BRAF* mutations in MSS CRCs are associated with poor survival [5, 7].

Altered cell metabolism is a hallmark of cancer maintaining malignant properties and sustained growth [8]. Studies on blood and stool samples have indicated differences in amino acid composition and lipid metabolism between CRC patients and healthy controls [9]. However, metabolite levels depend on the sample type analyzed [9]. Studies on metabolite levels in CRC tissue samples have remained few [10–12]. For example, increased glycolysis and lipid metabolism have been found in tumor tissue [13, 14]. None of these studies, however, correlated metabolic patterns with mutations in *BRAF*. In *BRAF-*mutated CRC cell lines, e.g. alterations in glycolysis and glutamine utilization have been reported [15].

Aberrant DNA methylation disturbs gene regulation and predisposes cells to malignant degeneration [16]. Several studies have linked mutations in *BRAF* with CIMP (see e.g. [5, 17]), while the mechanism has remained controversial. Studies have argued both that *BRAF* V600E mutations themselves drive methylation changes [18, 19] and that *BRAF* mutations alone are insufficient to induce DNA methylation [20, 21]. Tao et al. examined mouse colon-derived organoids and suggested that spontaneous, aging-like promoter hypermethylation suppresses senescence, activates stem cell signaling pathways, and creates permissive conditions for *BRAF-*driven tumorigenesis, favoring the survival of *BRAF-*mutated cells [21]. *BRAF* mutations have also been proposed to decrease the expression of TET enzymes [18], which are important regulators of DNA demethylation [22]. Fang et al. [23] linked *BRAF* mutations to increased methylation levels through increased activity of the MAFG transcriptional repressor. They suggested that MAFG binds to promoters of *MLH1* and other CIMP-related genes and recruits a corepressor complex including the de novo DNA methyltransferase DNMT3B, causing promoter hypermethylation.

Metabolic changes may be linked with epigenetic patterns. Tumors harboring mutations affecting the Tricarboxylic Acid Cycle (TCA cycle), such as fumarate hydratase deficient uterine leiomyomas and succinate dehydrogenase deficient gastrointestinal stromal tumors, show high methylation levels [24, 25]. Accumulating TCA cycle metabolites can inhibit the functioning of TET DNA demethylases, which may lead to hypermethylation. Thus, characterizing the metabolic patterns of *BRAF-*mutated CRCs could also shed light on the high methylation levels observed in these lesions.

Here, we have characterized the metabolomic, epigenetic, and transcriptomic patterns in MSS *BRAF* V600E mutated human CRCs to scrutinize the origin of their high DNA methylation levels.

## Materials and methods

### Samples

**Fig. 1:**
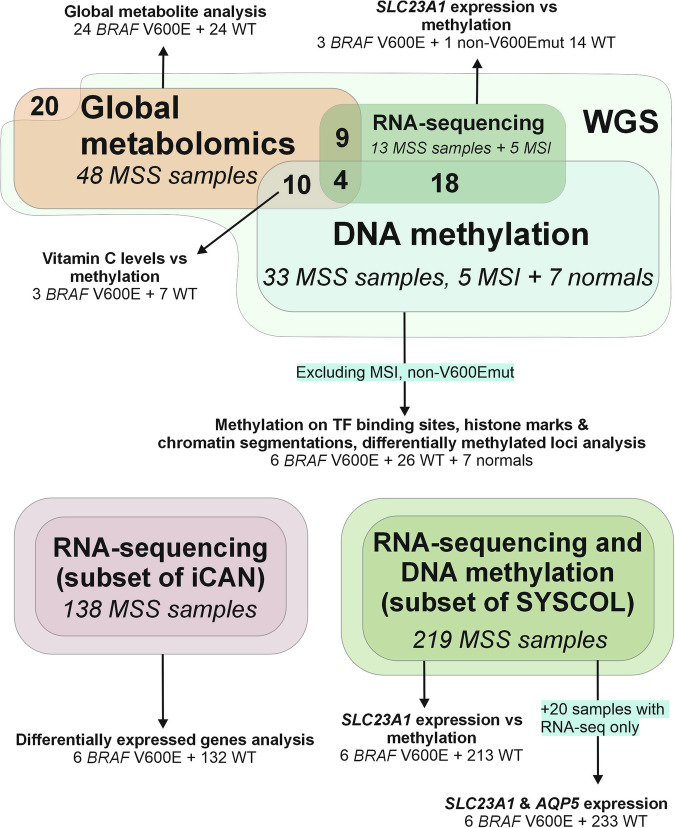
Data types, data overlaps and analyses presented in the study. MSI tumors were excluded from global metabolite analysis, linear models on methylation data, differentially methylated loci calling and differentially expressed genes analyses.

The study was conducted following the Declaration of Helsinki and was approved by National Institute for Health and Welfare and reviewed by the Ethics Committee of the Hospital District of Helsinki and Uusimaa (the latest permit numbers THL/1300/5.05.00/2019 and HUS/2509/2016). Samples were prospectively collected after informed consent from nine CRC treating hospitals in Finland [26–28]. Supplementary Table 1 summarizes the clinical characteristics and data types per sample. Samples in the differentially expressed genes analysis were collected within the iCAN Flagship project, which was reviewed by the HUS Ethical Committee and is executed based on a HUS research permit (4.5.2023 §38 (HUS/223/2023)), a Findata data permit (THL/1338/14.02.00/2022), and MTAs with Helsinki Biobank (HBP20210170) and Finnish Hematology and Registry Biobank FHRB (12.5.2022) (Supplementary Methods). Figure 1 summarizes the sample material used in the analyses.

### Global metabolite analysis

Global metabolomic profiling was performed by Metabolon Inc for fresh-frozen tissue samples from 24 MSS *BRAF* V600E mutated and 24 MSS non-mutated tumors. A minimum of 50 mg of sample material was cut from the tumor samples and stored at -80°C until shipment to Metabolon. Briefly, four independent platforms were utilized in the sample analysis: two separate reverse phase (RP)/UPLC-MS/MS methods with positive ion mode electrospray ionization (ESI), one analysis by RP/UPLC-MS/MS with negative ion mode ESI, and one analysis by HILIC/UPLC-MS/MS with negative ion mode ESI. All methods utilized a Waters ACQUITY ultra-performance liquid chromatography (UPLC) and a Thermo Scientific Q-Exactive high resolution/accurate mass spectrometer interfaced with a heated electrospray ionization (HESI-II) source and Orbitrap mass analyzer operated at 35,000 mass resolution. Multiple types of controls were applied in the analysis; a pooled matrix sample generated from each experimental sample served as a technical replicate; extracted water samples served as process blanks; and a cocktail of quality control standards were spiked into every analyzed sample, allowing instrument performance monitoring and aided chromatographic alignment. Compound identification was performed by comparing with library entries of purified standards or recurrent known entities. Peaks were quantified using the area under the curve. Two-way ANOVA was used to detect metabolite differences between *BRAF-*mutated and non-mutated tumors. The small sample size analyzed per group (*n* = 24) limits the statistical power of this analysis; thus, raw P-values from ANOVA tests are represented. Metabolites showing differential levels were analyzed with QIAGEN Ingenuity Pathway Analysis [29]; (QIAGEN Inc., https://digitalinsights.qiagen.com/IPA; v107193442).

### Whole-genome sequencing and somatic variant calling

The samples were processed as described previously [30, 31]. Briefly, DNA was isolated from fresh-frozen tissue samples and sequenced with the Illumina platform. Somatic variant calling was performed as described previously [30, 31] using the GRCh38 reference genome. *BRAF*-mutation status was extracted with BasePlayer v.1.0.2 [32] with minimum variant coverage of 4 reads and minimum allelic fraction of 10%. Twenty samples in the metabolite analysis lacked WGS data and the *BRAF* V600E mutation status was determined based on TaqMan assay, with positive cases confirmed with Sanger sequencing. MSI status was determined earlier [26–28, 33] (Supplementary Methods). Variant calling in the iCAN and SYSCOL cohorts is described in Supplementary Methods.

### CpG island methylator phenotype (CIMP)

CIMP status was defined as described previously [34]. Briefly, the methylation status of eight tumor suppressor genes (*CACNA1G, CDKN2A, CRABP1, IGF2, MLH1, NEUROG1, RUNX3, SOCS1*) was defined using a Methylation-Specific Multiplex Ligation-dependent Probe Amplification assay (MS-MLPA). Samples were classified as CIMP-high (5–8 genes methylated), CIMP-low (1–4 genes methylated), or CIMP-zero (0 genes methylated).

### Whole genome bisulfite sequencing

Whole genome bisulfite sequencing (WGBS) was performed for fresh-frozen normal colon (*n* = 7) and CRC (*n* = 38) samples. Library preparation and processing of the sequencing reads was performed as described previously [35]. Briefly, WGBS library preparations and Illumina sequencing were done as a service at BGI (BGI Tech Solutions Co., Ltd., China). Raw reads were processed with Trim Galore (v0.4.1) and aligned against the hg19/GRCh37 reference genome using Bowtie 2 (v2.3.0) [36]. Extraction of methylation calls was done with Bismark methylation extractor (v0.17.0).

### Statistical analysis of the methylation data and differentially methylated loci (DML) calling

A minimum read coverage of 6 was applied in all analyses. Average methylation values were calculated over multiple annotations on autosomal regions. Histone modifications, chromatin state segmentations and DNAse hypersensitivity regions from tissue samples of the normal human gastrointestinal tract and samples relevant to tumorigenesis were provided by the Roadmap Epigenomics [37] project (Supplementary Table 2). Hierarchical clustering was performed on 18,957 methylation measurements overlapping Flanking Bivalent TSS/Enhancers with R using dendextend v1.17.1 and dist() function with euclidean distances. An Ordinary Least Squares regression was performed explaining the mean methylation level on chromatin state segmentation, histone modification and DNAse hypersensitivity regions separately for each cell type and annotation type; *BRAF-*mutation status and genome-wide average methylation levels served as explanatory variables. Genomic annotations represented by ≥2 cell/tissue types entered the analysis. Transcription factor (TF) binding sites were obtained from CRC cell lines as described previously [38]. For TFs, average methylation level across all TFs, number of measurements overlapping TF binding sites and *BRAF* mutation status served as explanatory variables in linear models. Adjusted *P*-values were calculated using false discovery rate (FDR). DMLs were detected with the DSS [39] R package (v.2.28.0) callDML() function using delta=0.1 and p-threshold 0.01. DMLs were called in three separate analyses; comparing the *BRAF* V600E tumors against non-mutated tumors; *BRAF* V600E tumors against normals; and non-mutated tumors against normals. DML overlaps with promoter regions were identified using R-package annotatr (1.12.1). PANTHER Overrepresentation Test (Release 20230705) against Reactome database (v.77) was performed for genes with multiple hyper- or hypomethylated DMLs at promoter regions in the *BRAF* V600E vs. non-mutated tumor analysis. MSI tumors were excluded from linear models and DML analyses.

### RNA-sequencing and analysis of expression data

RNA-sequencing data used in the differentially expressed genes analysis was processed within iCAN (https://ican.fi/) with Illumina NovaSeq 6000 using the Illumina Stranded Total RNA kit and Dragen Bio-IT platform (Supplementary Methods). We selected MSS tumors by excluding samples showing an exceptionally high mutation count (Supplementary Fig. 1). Six *BRAF* V600E mutated and 132 non-mutated MSS CRCs entered the analysis. A likelihood ratio test was performed with DESeq2 (v.1.40.1) comparing a model ~RNA-sequencing batch + sex + *BRAF* mutation status to a reduced model ~RNA-sequencing batch + sex. Genes passing a minimum expression threshold of 10-read count sum over all tumors were included in the analysis. Genes with P-adj. < 0.05 were considered as significantly changed. *SLC23A1* expression counts were produced using plotCounts() function. Differentially expressed genes were analyzed with QIAGEN Ingenuity Pathway Analysis [29]; (QIAGEN Inc., https://digitalinsights.qiagen.com/IPA; v122103623).

### *SLC23A1* expression and correlation with DNA methylation

Correlation between *SLC23A1* expression [30] and methylation was performed for 18 samples undergone both RNA-sequencing and WGBS (Fig. 1) and validated in a cohort (SYSCOL [40]) comprising 219 MSS samples (Supplementary Methods). Correlation was calculated using Pearson correlation coefficients.

## Results

### Global metabolite analysis in MSS *BRAF* V600E mutated CRCs reveals decreased levels of vitamin C

**Fig. 2:**
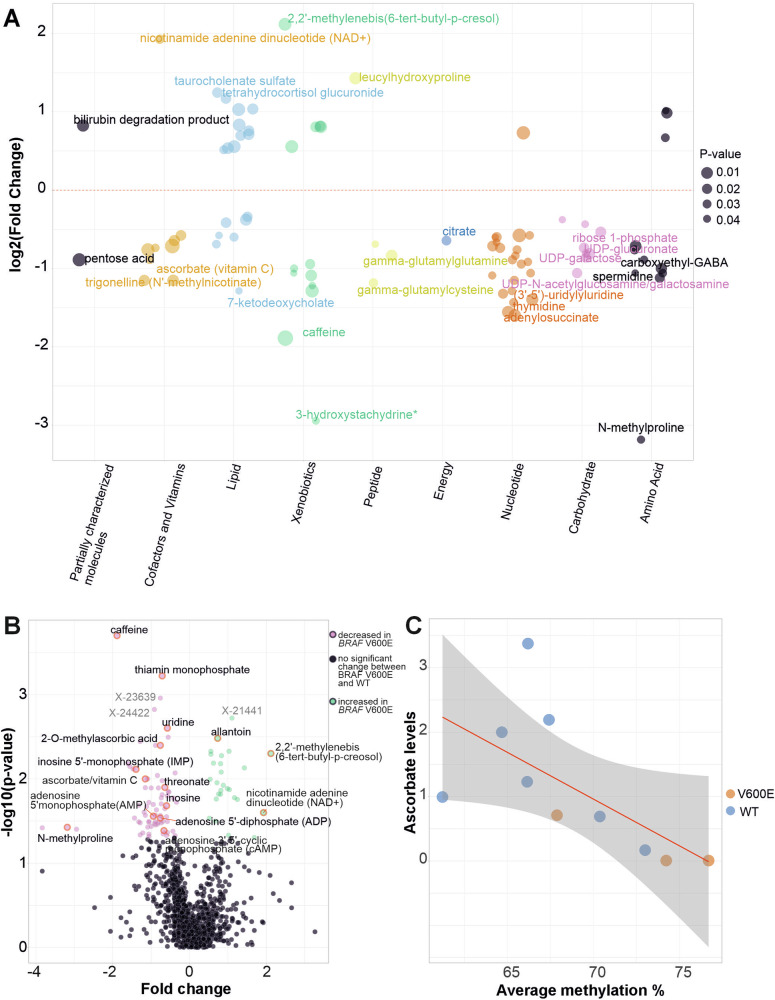
Results from metabolomic analysis. **A** Metabolic changes in MSS *BRAF* V600E tumors compared to MSS WT tumors for metabolites of known identity (*P* < 0.05). Each point depicts one metabolite, its size reflecting the significance of the result. Results are grouped by super pathway (X-axis). **B** Volcano plot for all 1126 metabolites. For readability, only selected metabolites are labeled in panels **A, B**. **C** Ascorbate levels vs. genome-wide DNA methylation levels (log-transformed ascorbate levels, r(8) = −0.84, *P* = 0.0022, Pearson correlation coefficient). The 95% confidence interval is shown in gray.

Global metabolite analysis was performed for fresh-frozen tissue samples from 24 MSS *BRAF* V600E mutated and 24 MSS non-mutated tumors (hereafter referred to as “WT”). The WT group consisted of 15 sporadic CRCs and nine cancers from patients diagnosed with inflammatory bowel disease (IBD). A total of 1126 compounds were detected, of which 1001 were named biochemicals of known identity. Of these, 99 showed significant (*P* < 0.05) alterations in *BRAF* V600E compared to WT tumors (27 upregulated, 72 downregulated; two-way ANOVA). Analysis of pathway enrichment [29] with metabolites depicted decreased activity of for example nucleotide catabolism and transport of vitamins, nucleosides and related molecules in *BRAF*-mutated CRCs (Supplementary Table 3). Most of the upregulated metabolites were lipids or xenobiotics (Fig. 2A). Multiple medium-chain fatty acids (pelargonate, cis-4-decenoate, (2 or 3)-decenoate, and 5-dodecenoate) showed increased levels in the *BRAF-*mutated tumors. We found no evidence for accumulation of TCA cycle metabolites in the *BRAF-*mutated tumors; citrate levels were decreased.

Significantly decreased levels of many purine metabolites were characteristic to *BRAF-*mutated tumors; xanthosine 5’-monophosphate, adenosine 5’-diphosphate, inosine, inosine 5’-monophosphate, adenosine 5’-monophosphate, adenosine 3’,5’-cyclic monophosphate, adenylosuccinate, adenosine, guanosine 5’-monophosphate and guanosine all showed significantly decreased levels in the *BRAF-*mutated tumors (Fig. 2A, B, Supplementary Table 4). Allantoin, a breakdown product of urate, was an exception and showed increased levels.

Nicotinamide adenine dinucleotide (NAD + ) levels were significantly increased in *BRAF-*mutated tumors (Fig. 2A, B), while its precursor nicotinamide showed decreased levels (Supplementary Table 4).

Among metabolites of known identity, the greatest fold change decrease in the *BRAF-*mutated tumors was observed for N-methylproline, a proline derivative obtained from diet, mainly from citrus fruits. In agreement, *BRAF-*mutated tumors displayed significantly decreased levels of vitamin C (ascorbate) and its metabolites threonate and 2-O-methylascorbic acid compared to WT tumors (Fig. 2A, B). Similar metabolite changes were observed in a comparison excluding IBD-CRCs from the WT group (Supplementary Table 4). Vitamin C is a cofactor for TET enzymes regulating DNA demethylation [41]. Low vitamin C levels associated with high DNA methylation levels (Fig. 2C, Supplementary Fig. 2).

### Expression data indicates downregulation of antioxidant action of vitamin C in MSS *BRAF* V600E mutated CRCs

To understand the gene expression changes induced by *BRAF*-mutations, we performed a differential expression analysis within the iCAN project (https://ican.fi/). A comparison of 6 MSS *BRAF* V600E mutated and 132 MSS WT tumors revealed 958 differentially expressed genes (P-adj. < 0.05), of which 773 were upregulated in *BRAF-*mutated tumors (Supplementary Table 5). *AQP5*, a marker for stemness in gastric cancer [42], was one of the most significantly upregulated genes.

**Fig. 3:**
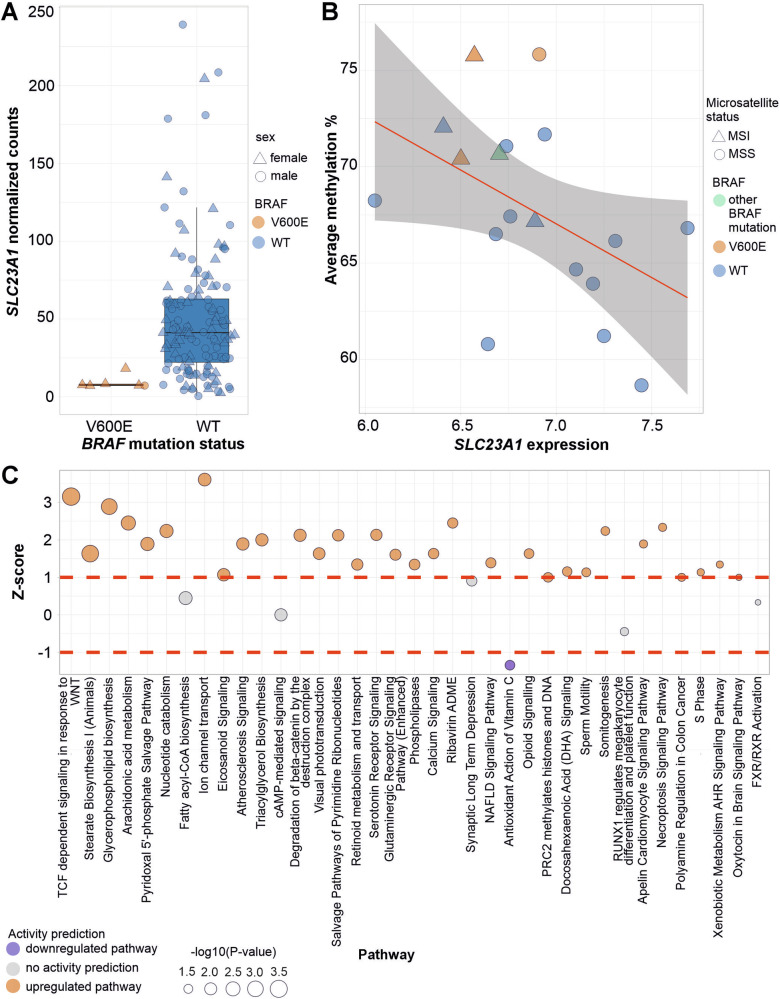
Gene expression analysis results. **A** SLC23A1 expression levels were decreased in MSS *BRAF* V600E tumors (Data: the iCAN cohort; P-adj. = 0.014). **B** SLC23A1 expression and genome-wide DNA methylation showed an inverse correlation (r(16) = −0.47, *P* = 0.051, Pearson correlation coefficient). The 95% confidence interval is shown in gray. **C** Ingenuity Pathway Analysis (IPA) results for the differentially expressed genes in MSS *BRAF* V600E vs. WT tumors. Significantly upregulated (orange) and downregulated (blue) canonical pathways sorted by *P*-value (larger circle reflects lower *P*-value). No activity prediction is made for pathways with Z-scores between -1 and 1 (gray). Pathways with *P* < 0.05 are shown. The z-score on the y-axis reflects predicted pathway activation (positive values) or inhibition (negative values). Most of the inferred pathways depicted upregulation in *BRAF*-mutated tumors. (Data: the iCAN cohort).

Compatible with metabolomics results, *SLC23* *A1*, which together with *SLC23A2* is responsible for C vitamin transport to cells [43], was downregulated in *BRAF-*mutated tumors (Fig. 3A). In a cohort of 18 CRC samples with both WGBS and RNA-sequencing data available (Fig. 1), we observed an inverse correlation between *SLC23A1* expression and DNA methylation levels, especially at flanking active TSSs (r(16) = −0.68, *P* = 0.0019) (Fig. 3B, Supplementary Fig. 3); low *SLC23A1* expression associated with high methylation levels. The inverse correlation between *SLC23A1* expression and methylation was further validated in the SYSCOL cohort [40] of 6 *BRAF-*mutated and 213 WT samples (Supplementary Fig. 4). High expression of *AQP5* was again characteristic for *BRAF-*mutated tumors in this cohort (Supplementary Fig. 4).

Analysis of pathway enrichment [29] for the differentially expressed genes indicated significant upregulation of, for example, TCF dependent signaling in response to WNT, glycerophospholipid biosynthesis, and ion channel transport in *BRAF* V600E tumors (Fig. 3C, Supplementary Table 6). Antioxidant action of vitamin C showed significantly reduced pathway activity in *BRAF* V600E tumors (Fig. 3C).

### Genome-wide DNA methylation changes by chromatin state and histone modification status

**Fig. 4:**
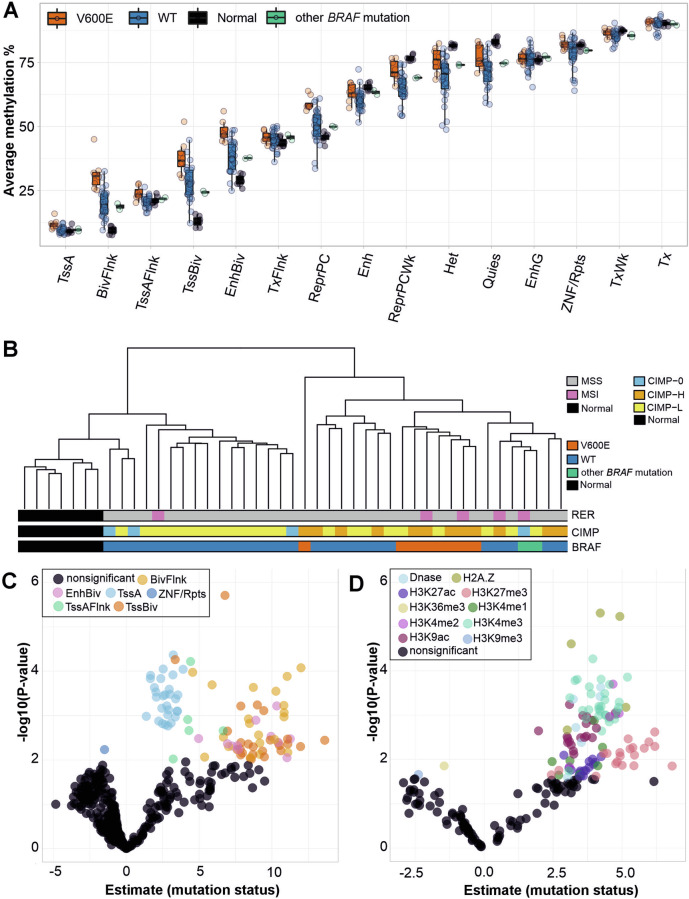
DNA methylation levels at different genomic annotations. **A** Average methylation percentage for all WGBS samples (MSS and MSI tumors, normal colon) at chromatin state segmentations obtained from colonic mucosa (E075) tissue in the Roadmap Epigenomics Project. TssA=Active Transcription Start Site (TSS), TssAFlnk=Flanking Active TSS, TxFlnk=Transcr. at gene 5’ and 3’, Tx=Strong transcription, TxWk=Weak transcription, EnhG=Genic enhancers, Enh=enhancers, ZNF/Rpts = ZNF genes & repeats, Het=Heterochromatin, TssBiv=Bivalent/Poised TSS, BivFlnk=Flanking Bivalent TSS/Enh, EnhBiv=Bivalent Enhancer, ReprPC=Repressed PolyComb, ReprPCWk=Weak Repressed PolyComb, Quies=Quiesent/low. Other BRAF mutation=other than V600E (*n* = 2). **B** Hierarchical clustering of all WGBS samples (MSS and MSI tumors, normal colon) based on methylation values collected from colonic mucosa BivFlnk areas (Roadmap Epigenomics Project). CIMP-0 = CIMP zero, CIMP-L = CIMP low, CIMP-H = CIMP high. **C** DNA methylation in MSS *BRAF* V600E tumors was compared to MSS WT tumors using linear models. Each point represents a result from the linear model from one cell/tissue type and chromatin state in the Roadmap Epigenomics Project data (list of all Roadmap annotations analyzed in Supplementary Table 2). A positive estimate indicates that *BRAF* mutations were associated with elevated methylation levels at these chromatin states. Results with P-adj. < 0.05 are colored by chromatin state; nonsignificant results are colored with black. **D** Similar linear models were applied to individual histone modifications and DNAse hypersensitivity regions from the Roadmap Epigenomics Project.

Several studies have suggested a link between *BRAF* mutations and CIMP, which is characterized by promoter hypermethylation [44]. We first examined DNA methylation patterns using a genome-wide approach. DNA methylation levels were analyzed from fresh-frozen tissue samples of 10 *BRAF-*mutated CRCs (7 MSS, 3 MSI), 28 WT CRCs (26 MSS, 2 MSI), and seven normal colon samples with WGBS. We utilized chromatin state segmentations provided by the Roadmap Epigenomics project (Supplementary Table 2) and characterized DNA methylation levels on these annotations. As expected, all samples showed higher methylation levels at heterochromatin compared to actively transcribed regions at chromatin segmentations obtained from colonic mucosa (Fig. 4A). *BRAF* V600E mutated tumors showed increased methylation values compared to WT tumors, especially at Flanking Bivalent transcription start site (TssBiv)/Enhancer marked (BivFlnk) chromatin (Fig. 4A). Unsupervised hierarchical clustering of tumors based on BivFlnk methylation values displayed tight clustering of tumors with *BRAF* V600E mutation (Fig. 4B).

*BRAF* mutations are associated with MSI [5, 17]. To understand methylation changes driven solely by *BRAF* V600E mutations, we limited further DNA methylation analyses to MSS tumors and excluded two samples showing mutations other than the V600E in the *BRAF* gene (resulting in 6 MSS *BRAF* V600E, 26 MSS WT tumors).

Next, we utilized linear models to identify genomic regions with methylation levels affected by *BRAF* V600E mutation status utilizing a broader set of annotations from tissue samples of the normal human gastrointestinal tract and samples relevant to tumorigenesis (Supplementary Table 2). MSS *BRAF* V600E tumors were compared to MSS WT tumors using linear models, where the average methylation level at each annotation separately was explained by the sample’s genome-wide average methylation and by *BRAF* mutation status. *BRAF* V600E mutation status was associated with elevated average methylation at transcription start sites (Active TSS, Flanking Bivalent TSS, Flanking Active TSS) when compared to WT tumors (Fig. 4C, Supplementary Fig. 5), suggesting promoter hypermethylation. We further analyzed methylation on histone mark annotations. MSS *BRAF* V600E tumors harbored elevated methylation levels especially at H3K4me3 marked chromatin, which is often found at active enhancers [45] (Fig. 4D, Supplementary Fig. 5). *BRAF* V600E mutations had the most significant effect on methylation levels at variant histone H2A.Z bound chromatin, a Roadmap Epigenomics annotation available for carcinoma cell lines and embryonic stem cells. This may suggest changes in H2A.Z loading, as H2A.Z occupancy and DNA methylation are anticorrelated [46]. Supplementary Fig. 5 summarizes results from comparing each MSS tumor group to normal colon.

### Elevated methylation levels at GLIS1 and CTCF but not MAFG binding sites in MSS *BRAF* V600E mutated CRCs

**Fig. 5:**
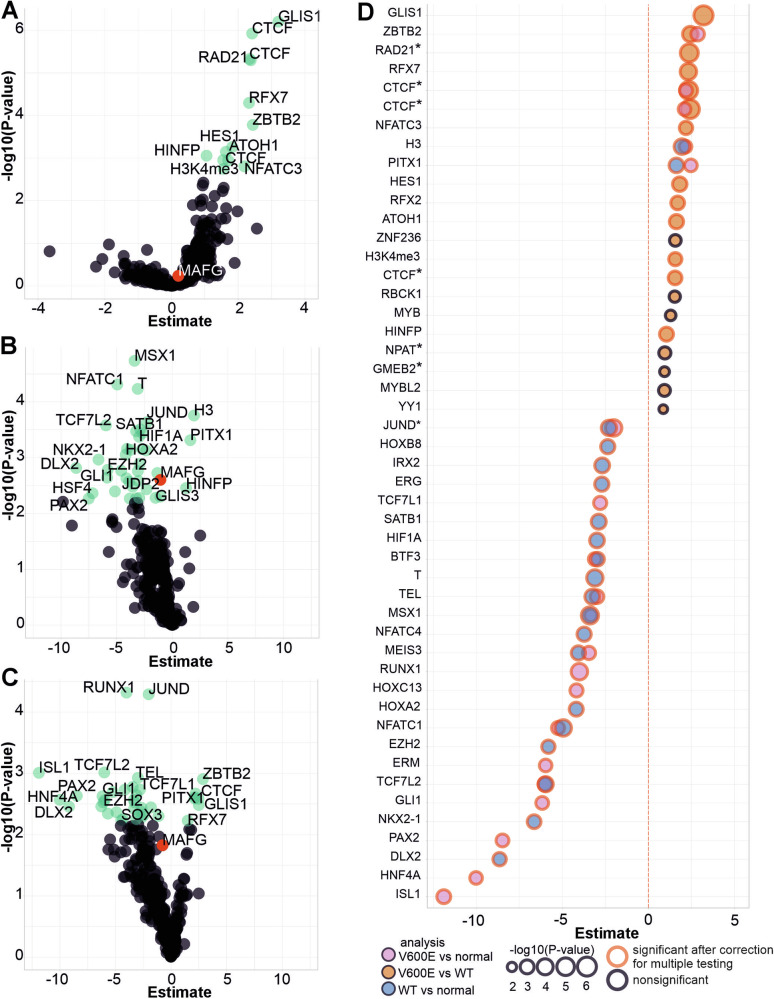
Transcription factor binding site methylation analysis. **A** *BRAF* V600E mutation was associated with high methylation levels e.g. at GLIS1 binding sites when MSS *BRAF* V600E tumors were compared to MSS WT tumors. MSS WT **B** and MSS *BRAF* V600E **C** tumors were compared separately to normal samples. Results with P-adj. < 0.05 are marked with green and nonsignificant results are colored with black. For readability, only a subset of the most significant results are labeled in panels **A–C**, please see Supplementary Table 7 for a comprehensive results list. MAFG is highlighted in red for visualization purposes. **D** Top 20 TFs with the most significant *p*-values from the three different models presented in **A–C** (red outline = P-adj. <0.05; * = several replicates of ChIP-sequencing data).

We analyzed the methylation levels on binding sites of 314 TFs and DNA binding proteins obtained from LoVo CRC cell line by ChIP-sequencing [38]. We first used the tumor samples (6 MSS *BRAF* V600E, 26 MSS WT) and again used linear models and explained the average binding site methylation for each TF separately by the sample’s average methylation level across all TF sites, the number of methylation measurements overlapping with the TF binding sites, and *BRAF* mutation status. After correcting for multiple testing, *BRAF* mutation status was most significantly associated with elevated average methylation levels at binding sites of GLIS1, CTCF, and RAD21 (Fig. 5A). We next compared the MSS *BRAF* V600E tumors to normals and the MSS WT tumors to normals (Fig. 5B, C). After correction for multiple testing, 26 shared factors were highlighted in both analyses: for example, tumor status was associated with decreased methylation at binding sites of TCF7L2 and EZH2, likely reflecting methylation changes characteristic for CRC genesis in general. Unexpectedly, both tumor groups had slightly decreased methylation levels at MAFG binding sites although according to Fang et al. [23], the binding of MAFG leads to the recruitment of DNMT3B, followed by methylation. Thirteen TFs showed statistical significance uniquely in the MSS *BRAF* V600E tumors vs. normals analysis. Four of these were also among the most significant results in the MSS *BRAF* V600E tumors vs. MSS WT tumors analysis: methylation levels of GLIS1, CTCF, RFX7, and ZBTB2 binding sites were all increased in the MSS *BRAF* V600E tumors (Fig. 5A, Supplementary Table 7). Elevated methylation at these TF binding sites may be of particular relevance for *BRAF* V600E CRCs.

### Differentially methylated loci show strong hypermethylation in MSS *BRAF* V600E mutated CRCs

We performed a genome-wide differentially methylated loci (DML) analysis comparing 6 MSS *BRAF* V600E tumors to 26 WT MSS tumors. In this analysis, we detected 629,117 DMLs out of which 96.4% were hypermethylated in MSS *BRAF* V600E tumors relative to MSS WT tumors (Table 1). We then performed a DML analysis comparing both tumor groups separately to a pool of normal samples. MSS WT tumors depicted an overall genomic hypomethylation pattern typically present in cancer tissues [16]. A much lower proportion of the DMLs were hypomethylated in the MSS *BRAF* V600E samples, revealing significantly more hypermethylation compared to the MSS WT tumors (*P* < 2.2*10^-16^, Chi-squared test).

**Table 1:** Number of differentially methylated loci in differentially methylated loci analyses.

Analysis	DMLs	Hyper	Hypo
*BRAF*-mutated vs. WT	629,117	606,651 (96,4%)	22,466 (3,57%)
*BRAF*-mutated vs. normals	1,326,676	559,455 (42,2%)	767,221 (57,8%)
WT vs. normals	4,883,781	337,007 (6.9%)	4,546,774 (93.1%)

### Hypermethylated DMLs are enriched at the promoters of neuronal system genes

**Fig. 6:**
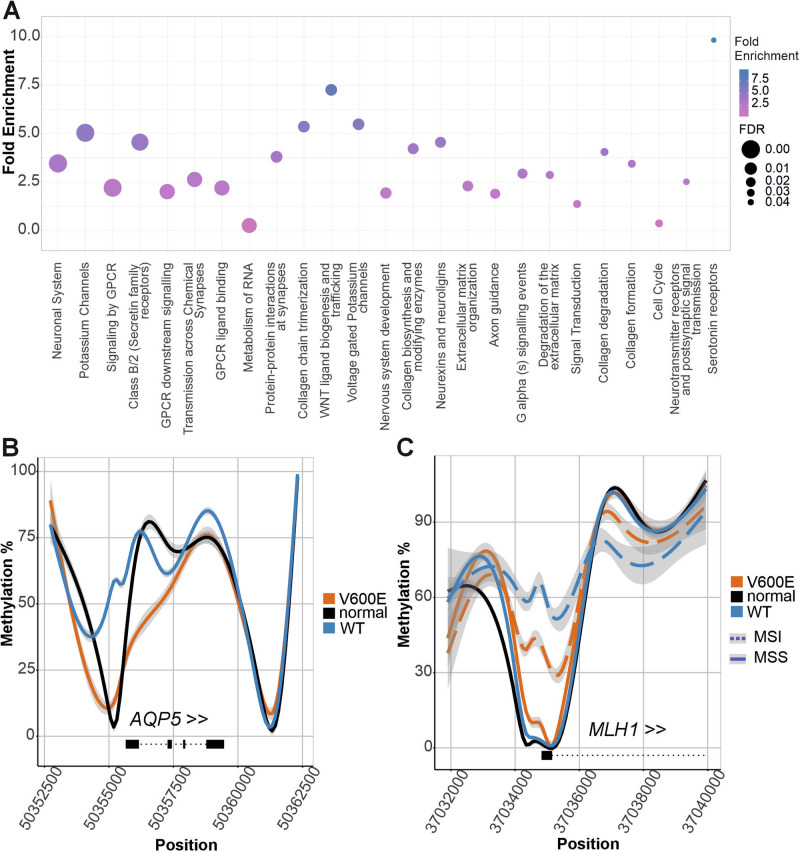
Methylation changes at gene promoters. **A** Gene set enrichment analysis of genes with >6 hyper-DMLs at their promoters in MSS *BRAF* V600E tumors. Results are sorted by FDR. **B** Pooled methylation levels across *AQP5* gene from 7 normal samples, 6 MSS *BRAF* V600E and 26 MSS WT samples. **C** Pooled methylation levels at the promoter of *MLH1* from 7 normal samples, 4 MSI samples (dashed lines, 2 *BRAF* V600E + 2 WT), and 32 MSS samples (solid lines, 6 *BRAF* V600E, 26 WT). *MLH1* promoter methylation is low in MSS *BRAF* V600E tumors. This, together with our observation that MAFG is not highlighted by the TF methylation analysis, suggests that a mechanism other than increased MAFG activity is likely to explain the overall hypermethylation pattern in MSS *BRAF*-mutated CRCs.

We annotated the DMLs against gene promoters (1 kbp upstream of TSS) to analyze methylation changes at promoter regions. In the MSS *BRAF* V600E tumors, 4158 genes showed at least 1 hypermethylated DML at their promoter when compared to MSS WT tumors. From all the genes showing promoter hypermethylation, the average number of hypermethylated loci at the promoter area in MSS *BRAF* V600E tumors was 6.2. Genes with >6 hyper-DMLs at promoter areas (*n* = 917 genes) showed strong enrichment in the Neuronal System (R-HSA-112316, P-adj. = 1.39E-11) (Fig. 6A). The promoter of *FOXL2NB* showed the highest number of hyper-DMLs (148 DMLs). In the MSS *BRAF* V600E tumors, 316 genes harbored at least 1 hypomethylated locus at the promoter region (average 1.9) when compared to MSS WT tumors. Overrepresentation test indicated no enrichments for genes having multiple (>=2) hypo-DMLs at their promoter (*n* = 36 genes). The promoter of *AQP5*, a gene upregulated in *BRAF* V600E tumors, showed an exceptionally high number of hypo-DMLs (73) (Fig. 6B).

MAFG has been suggested to bind to *MLH1* promoter and induce methylation in *BRAF-*mutated tumors [23]. According to the ChIP-sequencing data set utilized in our study, there is no MAFG binding site at the promoter region of *MLH1*, the nearest being located 0,5Mbp upstream. Methylation at the *MLH1* promoter area was strongly associated with MSI status (Fig. 6C).

## Discussion

Here, we performed a comprehensive molecular characterization of MSS *BRAF* V600E CRCs to study the link between *BRAF* mutations, tumor metabolism and DNA methylation. Our global metabolomics analysis revealed decreased levels of vitamin C and its metabolites in *BRAF* V600E compared to WT tumors. Pathway analysis of differentially expressed genes suggested significant downregulation of antioxidant action of vitamin C in *BRAF* V600E tumors, providing further evidence for the role of vitamin C in these lesions. Importantly, decreased levels of vitamin C provide a potential link between high methylation levels and *BRAF* mutations. Our genome-wide methylation analysis indicated that *BRAF* V600E mutations are associated with elevated methylation levels especially at transcription start sites and at H3K4me3 and H2A.Z bound chromatin.

Vitamin C is an important cofactor of TET enzymes, which have a crucial role in regulation of DNA demethylation [41]. Vitamin C has been shown to boost DNA demethylation in many cell lines, including *BRAF*-mutated cancer cell lines [47, 48]. Intriguingly, we observed decreased expression of the vitamin C transporter *SLC23A1* in *BRAF* V600E tumors and a negative correlation between *SLC23A1* expression and DNA methylation levels in two cohorts of CRCs. Vitamin C poor diet or low transportation of vitamin C to cells could facilitate the development of *BRAF* V600E tumors by reducing TET DNA demethylation activity, which could be reflected in the high methylation levels in *BRAF* V600E CRCs. Tao et al. have suggested that aging-like, spontaneous hypermethylation produces permissive conditions for *BRAF-*mutated cells [21]. We propose that similar permissive conditions could also be induced by decreased TET activity due to low vitamin C levels. Moreover, vitamin C has been shown to selectively kill *BRAF* and *KRAS-*mutated colorectal cancer cells [49], further suggesting that low vitamin C levels may facilitate growth of *BRAF-*mutated tumors. Recently, higher total vitamin C intake after CRC diagnosis was associated with lower CRC-specific mortality in patients with *KRAS* or *BRAF* mutated tumors but not in individuals with *KRAS/BRAF* wild type tumors [50]. Vitamin C is water-soluble, indicating that its metabolic levels may only reflect recent vitamin C intake. However, the observed negative correlation between vitamin C levels and DNA methylation in our data implies a longer-term difference in vitamin C intake or metabolism in *BRAF-*mutated tumors.

Decreased vitamin C levels may indicate reduced antioxidant capacity. Our data suggests that this reduction may be partly compensated by the antioxidant action of urate in *BRAF* V600E tumors. During oxidative stress, the reaction between urate and superoxide or hydroxyl radicals results in the formation of allantoin. Thus, allantoin levels can serve as a biomarker for oxidative stress [51]. We observed increased allantoin levels in *BRAF* V600E CRCs, which may indicate elevated reaction rate between urate and reactive oxygen species. Levels of urate were not significantly changed. Nevertheless, as urate is the end product of purine metabolism, our observation on decreased purine metabolites in *BRAF* V600E CRCs may be linked with increased consumption of urate in these antioxidant reactions. Intriguingly, vitamin C supplementation has been shown to reduce allantoin levels in plasma [52], suggesting that decreased vitamin C levels in *BRAF* V600E CRCs may indeed be linked with the observed increase of allantoin levels.

Methylation analyses depicted elevated methylation levels at the binding sites of GLIS1 and CTCF in *BRAF* V600E CRCs. Importantly, MAFG was not highlighted in our analyses. Thus, a mechanism other than the previously proposed increased MAFG activity [23] is likely to explain hypermethylation in MSS *BRAF* V600E CRCs. GLIS1 facilitates induction of pluripotency in the generation of induced pluripotent stem cells [53, 54] through an interplay between epigenetic and metabolomic factors. GLIS1 upregulates glycolytic activity by modulating histone acetylation and lactylation during reprogramming. Knockdown of GLIS1 decreases both glucose uptake and lactate secretion [53]. Despite the potential decrease in GLIS1 activity in *BRAF* V600E tumors, our metabolite data didn’t reveal clear changes in these processes. Methylation changes at the binding sites of CTCF may reflect decreased CTCF occupancy in *BRAF* V600E tumors as DNA methylation can inhibit the binding of CTCF [55, 56]. Changes in CTCF binding have been associated with DNA methylation in multiple cancer types, including gastrointestinal stromal tumors, where CTCF binding site hypermethylation resulted in loss of CTCF binding [57]. Intriguingly, 5-hydroxymethylcytosine and 5-formylcytosine, intermediates of TET-mediated oxidative DNA demethylation, have been suggested to facilitate CTCF binding [57]. Thus, reduced TET activity due to low vitamin C levels in *BRAF* V600E tumors could affect CTCF binding also through reduced 5-hydroxymethylcytosine and 5-formylcytosine.

High expression of *AQP5* and hypomethylation at its promoter region suggest that this gene has biological relevance in *BRAF* V600E tumors. In addition to water, aquaporins facilitate transportation of other small molecule-derived solutes, such as H2O2, and thus also play a role in the regulation of antioxidant activity and generation of ROS [58]. *AQP5* expression is usually detected in CRC, but not in the corresponding normal tissues [59]. Overexpression of *AQP5* was shown to induce cell proliferation through activation of ERK phosphorylation in CRC cells [60]. Thus, the high expression of *AQP5* in *BRAF* V600E tumors could facilitate oncogenic RAS-RAF-MEK-ERK-MAPK signaling promoted by V600E. Recent studies show that *AQP5* is enriched for stem cells in gastric cancer [42] and early gastric cardia adenocarcinoma [61]. Stem cells expressing *AQP5* were shown to act as a source for WNT-driven, invasive gastric cancer in mouse models [42]. Results from gastric cardia adenocarcinoma indicate that nicotinamide N-methyltransferase (NNMT) is enriched in *AQP5* expressing stem cells and that their stemness is maintained by NNMT-mediated nicotinamide metabolism, which causes reduction of H3K27 trimethylation and activation of WNT signaling [61]. Thus, it is possible that *BRAF* V600E CRCs depict a WNT activation mechanism similar to that observed in gastric cancer. Our metabolomics analysis identified alterations in levels of nicotinamide adenine dinucleotide and nicotinamide in MSS *BRAF* V600E tumors, yet the expression level of *NNMT* was similar to WT tumors.

A small sample size precludes the usage of potentially important clinical cofactors, such as sex and age, in statistical analyses. The data layers analyzed were largely non-overlapping. However, non-overlapping data sets increase the total number of MSS *BRAF* V600E tumors analyzed in this study (*n* = 39) and provide strong support for the observations highlighted by different data layers.

Taken together, our results suggest that high methylation levels in MSS *BRAF* V600E tumors may be linked to decreased vitamin C levels, and consequent reduced TET DNA demethylase activity. As vitamin C is a cofactor for TET DNA-demethylase enzymes, defects in vitamin C metabolism or vitamin C intake could provide a direct link to increased methylation levels observed in *BRAF-*mutated tumors. Our results should fuel further work to validate the findings and to analyze whether vitamin C poor diet and defects in cellular vitamin C intake play a role in the genesis of *BRAF-*mutated tumors.

## Supplementary information

Supplementary Material

iCAN banner author list

Supplementary Table 1

Supplementary Table 2

Supplementary Table 3

Supplementary Table 4

Supplementary Table 5

Supplementary Table 6

Supplementary Table 7

## Data Availability

Genome-wide somatic single nucleotide variant calls (GRCh38) are deposited in the EGA database under accession code EGAS00001004710. Access to iCAN RNA-sequencing data can be applied from Helsinki BioBank. SYSCOL RNA-sequencing data is available in the EGA database under accession code EGAS00001002376.
