# Supplementary material for: Comprehensive metabolomic and epigenomic characterization of microsatellite stable BRAF-mutated colorectal cancer
Source: Oncogene. 2025 Mar 18;44(22):1718–30. doi: 10.1038/s41388-025-03326-y (PMC12122379; doi:10.1038/s41388-025-03326-y)
Supplement: Supplementary file 1 — Supplementary Material [file 41388_2025_3326_MOESM1_ESM.pdf]

Supplementary Material for Comprehensive metabolomic and epigenomic characterization of microsatellite stable *BRAF*-mutated colorectal cancer

Aurora Taira[1,2], Mervi Aavikko[1,2,3], Riku Katainen[1,2,3], Eevi Kaasinen[1,2], Niko Välimäki[1,2], Janne Ravantti[1,2,4], Ari Ristimäki[2,5], Toni T. Seppälä[2,6,7,8,9], Laura Renkonen-Sinisalo[2,6], Anna Lepistö[2,6], Kyösti Tahkola[10], Anne Mattila[10], Selja Koskensalo[11], Jukka-Pekka Mecklin[12,13], Jan Böhm[14], Jesper Bertram Bramsen[15], Claus Lindbjerg Andersen[15], Kimmo Palin[1,2,9], Kristiina Rajamäki[1,2], Lauri A. Aaltonen[1,2,9*] & iCAN[**]

* Corresponding author. Contact: lauri.aaltonen@helsinki.fi, Tel: +358-2941-25595

** A list of authors and their affiliations appears at the end of the paper.

1 Medicum/Department of Medical and Clinical Genetics, University of Helsinki, Helsinki, 00014, Finland

2 Applied Tumor Genomics Research Program, Research Programs Unit, University of Helsinki, Helsinki, 00014, Finland

3 Institute for Molecular Medicine Finland (FIMM), HiLIFE, University of Helsinki, Helsinki, Finland

4 Molecular and Integrative Biosciences Research Programme, Faculty of Biological and Environmental Sciences, University of Helsinki, FI-00014, Helsinki, Finland

5 Department of Pathology, HUSLAB, HUS Diagnostic Center, University of Helsinki and Helsinki University Hospital, Helsinki, 00014, Finland

6 Department of Surgery, Helsinki University Central Hospital, Hospital District of Helsinki and Uusimaa, Helsinki, 00290, Finland

7 Department of Gastroenterology and Alimentary Tract Surgery, Tampere University Hospital and TAYS Cancer Centre, 33520, Tampere, Finland

8 Faculty of Medicine and Health Technology, Tampere University, Tampere, 33100, Finland

9 iCAN Digital Precision Cancer Medicine Flagship, University of Helsinki, Helsinki, 00014, Finland.

10 Department of Surgery, The Wellbeing Services of Central Finland, Hoitajatie 1, 40620, Jyväskylä, Finland

11 The HUCH Gastrointestinal Clinic, Helsinki University Central Hospital, Helsinki, 00280, Finland

12 Department of Education and Research, The Wellbeing Services of Central Finland, Hoitajatie 1, 40620, Jyväskylä, Finland.

13 Department of Sport and Health Sciences, University of Jyväskylä, 40014, Jyväskylä, Finland

14 Department of Pathology, The Wellbeing Services of Central Finland, Hoitajatie 1, 40620, Jyväskylä, Finland

15 Department of Molecular Medicine, Aarhus University Hospital, DK-8200, Aarhus, Denmark; Department of Clinical Medicine, Aarhus University, DK-8200, Aarhus, Denmark.

List of Supplementary Tables 2
Supplementary Methods 3
Determination of microsatellite instability status 3
Information on the iCAN project 3
iCAN samples and data processing 3
Analysis of the SYSCOL methylation and expression data 4
Data visualization 4
References for Supplementary Methods 4
Supplementary Figure 1. Somatic variant counts in iCAN RNA-sequencing samples. 6
Supplementary Figure 2. Ascorbate levels vs DNA methylation. 7
Supplementary Figure 3. Vitamin C transporter expression levels compared with DNA methylation levels. 9
Supplementary Figure 4. AQP5 and SLC23A1 expression and comparison of SLC23A1 expression with DNA methylation in the SYSCOL cohort. 11
Supplementary Figure 5. Comparison of different linear model results. 13
iCAN consortium author information 15

List of Supplementary Tables

Supplementary Tables are given as separate files.

Supplementary Table 1. Clinical characteristics and data types available per sample.
Supplementary Table 2. Information on Roadmap Epigenomics datasets utilized in the analyses.
Supplementary Table 3. Results from Ingenuity Pathway Analysis (IPA) for metabolites.
Supplementary Table 4. Results from the metabolite analysis.
Supplementary Table 5. Results from the differentially expressed genes analysis.
Supplementary Table 6. Results from Ingenuity Pathway Analysis (IPA) for differentially expressed genes.
Supplementary Table 7. Results from the transcription factor binding site methylation analysis.

The list of iCAN authors is given in the **iCAN banner authorship list**.

Supplementary Methods

Determination of microsatellite instability status

For samples processed in our laboratory, the microsatellite instability (MSI) status was determined earlier as described in[1–4]. Briefly, in our sample collection microsatellite instability has been evaluated with radioactive labeling techniques, fluorescence-based PCR methods or fragment analysis. With radioactive labeling techniques, seven markers (D5S404, D17S787, D5S346, D1S216, D11S904, D10S197, and TP53) were utilized to evaluate microsatellite instability. Sample has been defined as MSI if 2/7 markers are unstable and MSS if none of the markers were unstable, given that at least five markers had been successfully analyzed. If 1/7 markers were unstable, more markers (DCC, D13S175, D7S519, D20S100, D15S120, D2S136, and D14S79) have been analyzed to achieve at least 10 evaluated markers in total. If at least one of the extra markers was unstable, a sample was called MSI. For samples where a fluorescence-based PCR method was utilized, 16 markers (D8S254, MYC, NM23, D5S346, TP53, D1S228, D8S261, D7S496, D8S137, DCC, D7S501, MCC, D5S318, D1S507, D19S394, and RB1) were utilized. In this case, if at least 30% of the alleles tested are unstable, a sample is called MSI. In case of fragment analysis, Bethesda panel of five markers (BAT25, BAT26, D5S346, D17S250, and D2S123) has been utilized. If at least 2/5 markers showed instability, the sample was called MSI.

Information on the iCAN project

RNA-sequencing data utilized in the differentially expressed genes analysis was analyzed within The iCAN Flagship project. More information on the iCAN project can be found at www.ican.fi. The iCAN Flagship project was reviewed by the HUS Ethical Committee and is executed based on a HUS research permit (4.5.2023 §38 (HUS/223/2023), a Findata data permit (THL/1338/14.02.00/2022), and MTAs with Helsinki Biobank (HBP20210170) and Finnish Hematology and Registry Biobank FHRB (12.5.2022). The iCAN project is carried out entirely in the HUS Acamedic environment, which is authenticated as one of the national safe data environments by Findata. Any results leveraging data obtained under a data permit (Secondary Health Care Act) is provided to Findata for confirmation of anonymity prior to export.

iCAN samples and data processing

Samples were scored as CRC after histopathological review. Fresh frozen tumor tissue samples from 219 CRC patients and corresponding normal samples from peripheral blood entered whole-exome sequencing (WES) with Illumina NovaSeq 6000 with target kit Twist Exome 2.0 plus Comp Exome spike-in (total target length 37,741,012 bp) and read length 2x101 bp. WES was performed aiming at >90% of target covered with >30x for normal and >95% of target covered with >100x for tumors. RNA-sequencing was performed for 165 CRC samples with Illumina NovaSeq 6000 using the Illumina Stranded Total RNA kit (with Ribo-Zero plus) aiming at >100 million read pairs at 2x101 bp read length. WES and RNA-sequencing data were analyzed using Illumina Dragen Bio-IT platform (Dragen host software version V.07.021.645.4.0.3, Bio-IT Processor v.0x18101306). The reference genome used was GRCh38. The resulting WES variant calls were utilized to identify samples carrying *BRAF* V600E mutations and to identify samples with exceptionally high mutation counts. BRAF-mutation status from the WES variant files was extracted using BasePlayer version 1.0.2[5]. WES variant counts were produced with bcftools version 1.18. We considered samples with high mutation load as microsatellite unstable and excluded these samples from the differential expression analysis focusing on microsatellite stable tumors (**Supplementary Figure 1**). Samples showing mutations other than the V600E in the

BRAF gene were excluded from the differentially expressed genes analysis. Thus, 138 RNA-sequenced tumor samples entered the differentially expressed genes analysis.

Analysis of the SYSCOL methylation and expression data

SYSCOL RNA-sequencing and methylation data (Infinium HumanMethylation450 BeadChip) were preprocessed as in [6]. *BRAF*-mutation status was determined[6] by singleplex PCRs using LightScanner Master Mix and LightScanner analysis (Idaho Technology). Average DNA methylation levels on different annotations was defined as the average methylation of probes overlapping each genomic annotation (First Exons, 3'UTRs, 5'UTRs, Gene Bodys, transcription start site upstream sequences 1500bp and 200bp and other regions). Probes overlapping with multiple annotations were excluded. Analysis was limited to autosomal regions. Transcriptome quantification was performed using Tophat2[7] and Cufflink[8] as described in [6]. The correlation between *SLC23A1* expression and average DNA methylation values and the normalized expression values for *SLC23A1* and *AQP5* were visualized using ggplot2 R-package[9].

Data visualization

Smoothed methylation values around *AQP5* and *MHL1* were produced using R-package ggplot2 (v.3.3.5) with geom_smooth() function. Graphical abstract was created using BioRender (BioRender.com).

References for Supplementary Methods

1 Aaltonen LA, Salovaara R, Kristo P, Canzian F, Hemminki A, Peltomäki P *et al.* Incidence of hereditary nonpolyposis colorectal cancer and the feasibility of molecular screening for the disease. *N Engl J Med* 1998; **338**: 1481–1487.

2 Salovaara R, Loukola A, Kristo P, Kääriäinen H, Ahtola H, Eskelinen M *et al.* Population-based molecular detection of hereditary nonpolyposis colorectal cancer. *J Clin Oncol* 2000; **18**: 2193–2200.

3 Tanskanen T, Gylfe AE, Katainen R, Taipale M, Renkonen-Sinisalo L, Mecklin J-P *et al.* Exome sequencing in diagnostic evaluation of colorectal cancer predisposition in young patients. *Scand J Gastroenterol* 2013; **48**: 672–678.

4 Kondelin J, Martin S, Katainen R, Renkonen-Sinisalo L, Lepistö A, Koskensalo S *et al.* No evidence of EMAST in whole genome sequencing data from 248 colorectal cancers. *Genes Chromosomes Cancer* 2021; **60**: 463–473.

5 Katainen R, Donner I, Cajuso T, Kaasinen E, Palin K, Mäkinen V *et al.* Discovery of potential causative mutations in human coding and noncoding genome with the interactive software BasePlayer. *Nat Protoc* 2018; **13**: 2580–2600.

6 Bramsen JB, Rasmussen MH, Ongen H, Mattesen TB, Ørntoft M-BW, Árnadóttir SS *et al.* Molecular-Subtype-Specific Biomarkers Improve Prediction of Prognosis in Colorectal Cancer. *Cell Rep* 2017; **19**: 1268–1280.

7 Kim D, Pertea G, Trapnell C, Pimentel H, Kelley R, Salzberg SL. TopHat2: accurate alignment of transcriptomes in the presence of insertions, deletions and gene fusions. *Genome Biol* 2013; **14**: R36.

8 Trapnell C, Williams BA, Pertea G, Mortazavi A, Kwan G, van Baren MJ *et al.* Transcript assembly and quantification by RNA-Seq reveals unannotated transcripts and isoform switching during cell differentiation. *Nat Biotechnol* 2010; **28**: 511–515.

9 Wickham H. *ggplot2: Elegant Graphics for Data Analysis*. Springer Science & Business Media, 2009.

Supplementary Figure 1. Somatic variant counts in iCAN RNA-sequencing samples.

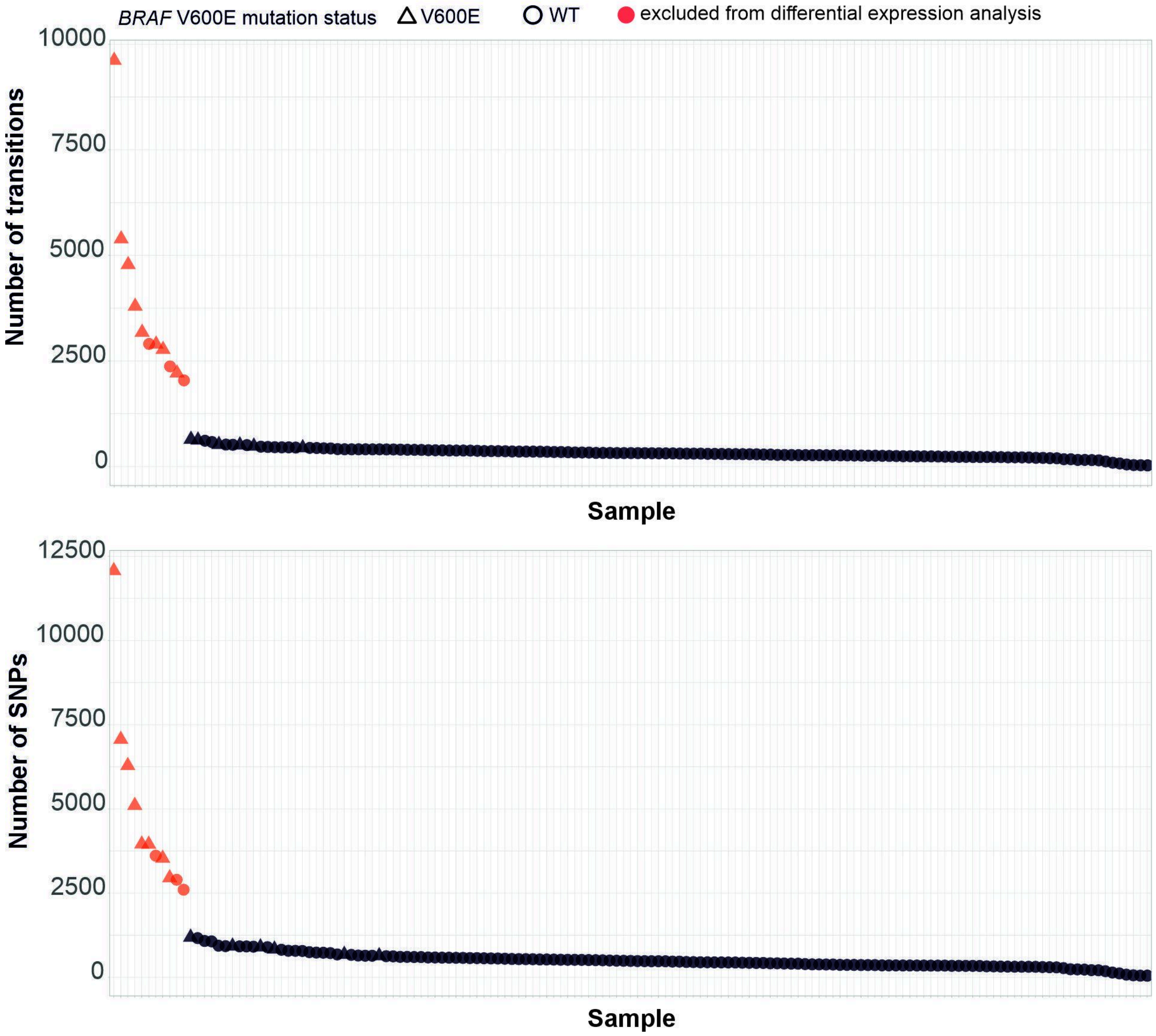

Supplementary Figure 1. Number of somatic transitions and SNPs in iCAN RNA-sequencing colorectal cancer sample collection (n=165 CRCs). Variant counts are based on whole-exome sequencing. Samples marked with red were excluded from differentially expressed genes analysis in order to focus on microsatellite stable tumors (See: Main text methods; Supplementary Methods).

Supplementary Figure 2. Ascorbate levels vs DNA methylation.

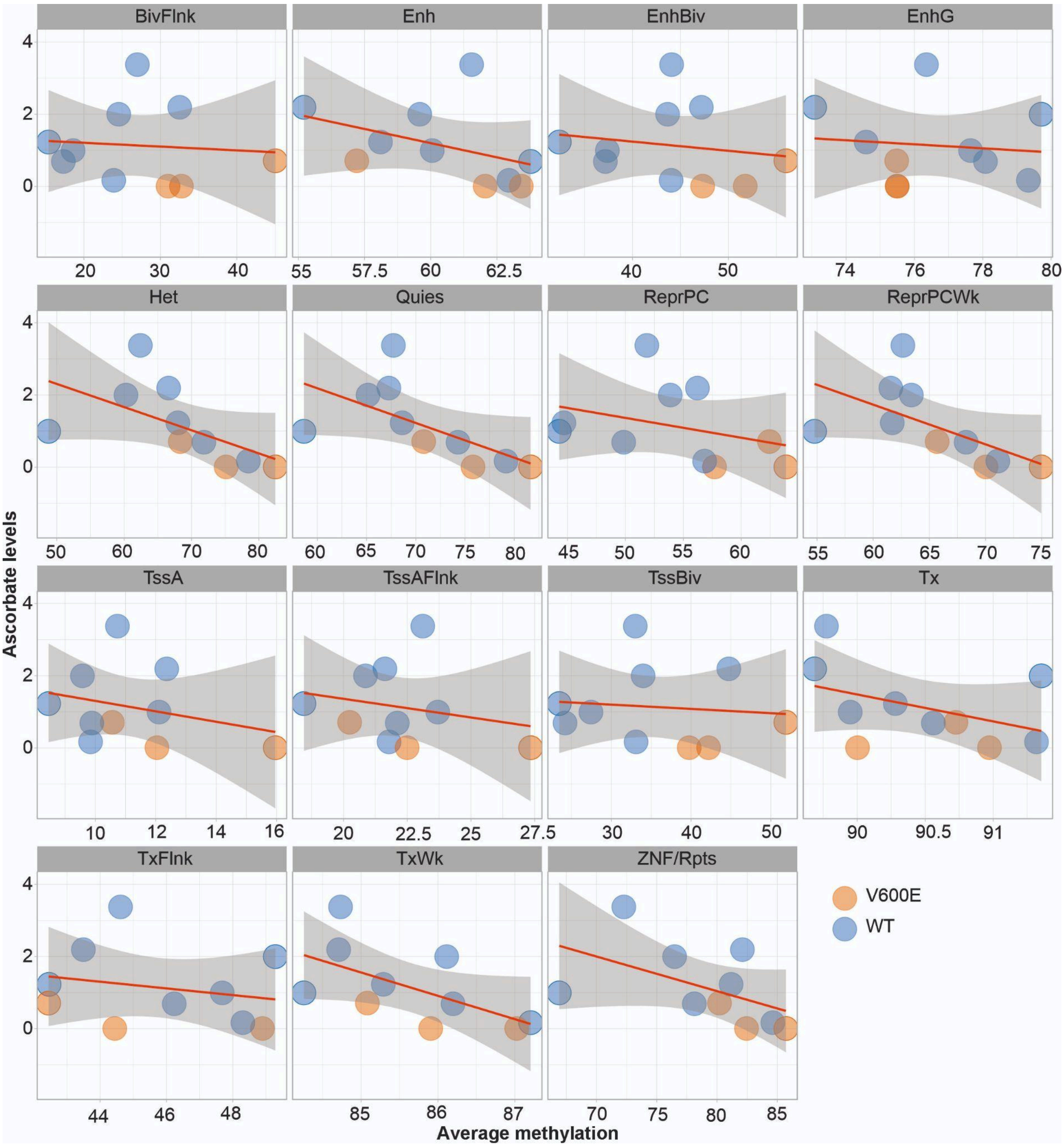

Supplementary Figure 2. Ascorbate levels vs DNA methylation levels in 3 MSS BRAF V600E tumors and 7 MSS WT tumors at different genomic regions originating from Roadmap epigenomics colonic mucosa annotations. The 95% confidence interval is shown in gray. Note the different scales on X-axis. Abbreviations and the Pearson correlation coefficients are listed below. Correlation was calculated using Pearson correlation coefficients analyzing the ascorbate counts on the natural log scale.

TssA=Active Transcription Start Site (TSS), r(8)=-0.62, P=0.056, 95% CI [-0.90; 0.018]

TssAFlnk=Flanking Active TSS, r(8)=-0.56, P=0.091, 95% CI [-0.88; 0.11]

TxFlnk=Transcr. at gene 5' and 3', r(8)=-0.25, P=0.48, 95% CI [-0.76; 0.45]

Tx=Strong transcription, r(8)=-0.22, P=0.55, 95% CI [-0.74; 0.48]

TxWk=Weak transcription, r(8)=-0.61, P=0.062, 95% CI [-0.89; 0.036]
EnhG=Genic enhancers, r(8)=-0.093, P=0.80, 95% CI [-0.57; 0.68]
Enh=enhancers, r(8)=-0.54, P=0.11, 95% CI [-0.87; 0.14]
ZNF/Rpts=ZNF genes & repeats, r(8)=-0.57, P=0.086, 95% CI [-0.88; 0.095]
Het=Heterochromatin, r(8)=-0.70, P=0.025, 95% CI [-0.92; -0.12]
TssBiv=Bivalent/Poised TSS, r(8)=-0.28, P=0.44, 95% CI [-0.77; 0.43]
BivFlnk=Flanking Bivalent TSS/Enh, r(8)=-0.26, P=0.47, 95% CI [-0.76; 0.44]
EnhBiv=Bivalent Enhancer, r(8)=-0.39, P=0.27, 95% CI [-0.82; 0.32]
ReprPC=Repressed PolyComb, r(8)=-0.55, P=0.097, 95% CI [-0.88; 0.12]
ReprPCWk=Weak Repressed PolyComb, r(8)=-0.76, P=0.012, 95% CI [-0.94; -0.24]
Quies=Quiesent/low r(8)=-0.74, P=0.015, 95% CI [-0.93; -0.21]

Supplementary Figure 3. Vitamin C transporter expression levels compared with DNA methylation levels.

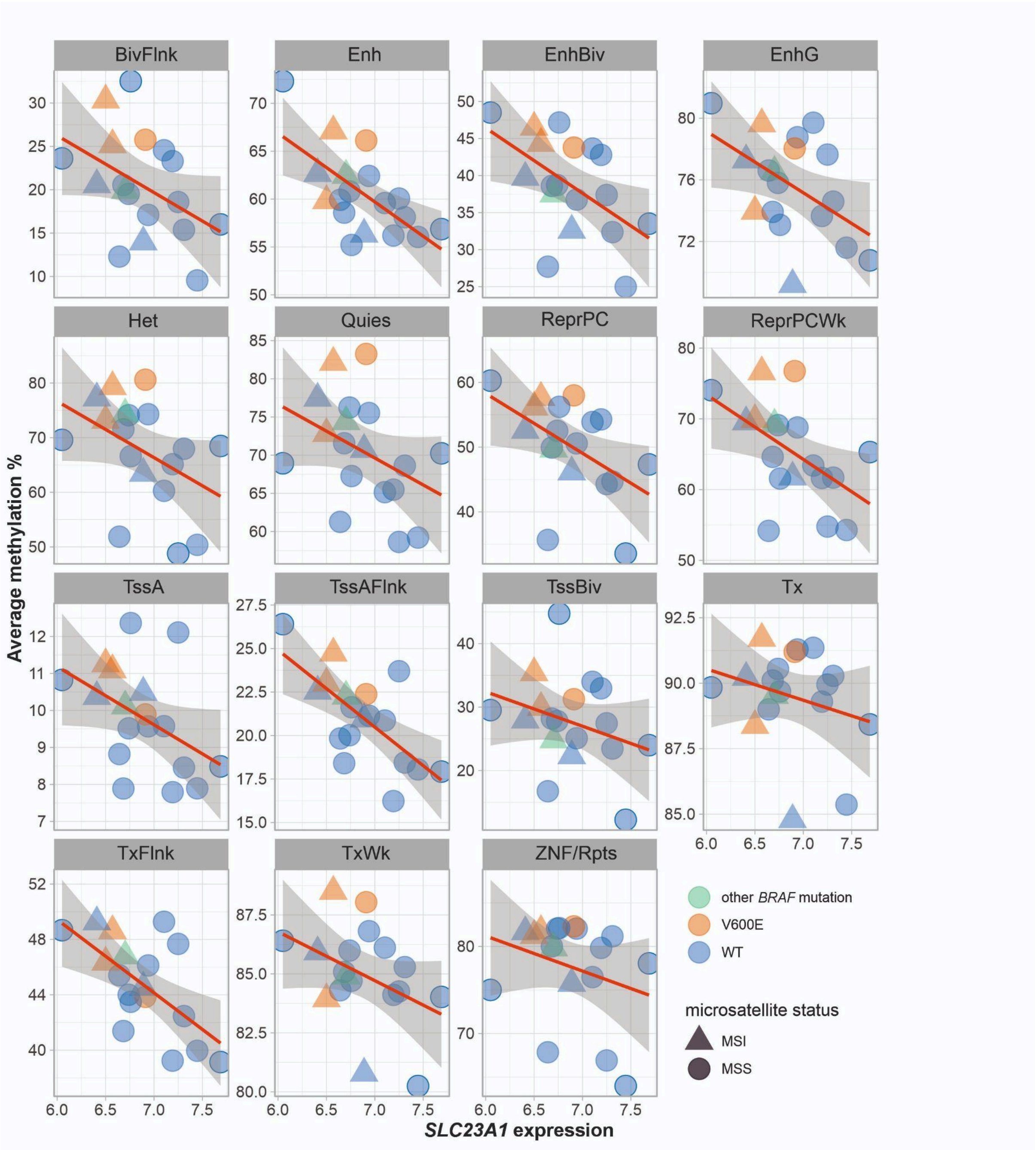

Supplementary Figure 3. Vitamin C transporter gene SLC23A1 expression vs average DNA methylation levels at different genomic regions originating from Roadmap epigenomics colonic mucosa annotations.The 95% confidence interval is shown in gray. Data is from a collection of 18 samples containing 3 BRAF V600E mutated samples, 1 sample with another BRAF mutation (Arg437Stop) and 14 WT samples. In this dataset, all BRAF-mutated samples were MSI. Note different scales on Y-axis. Abbreviations and the Pearson correlation coefficients are listed below.
TssA=Active Transcription Start Site (TSS), , r(16)=-0.45, P=0.061, 95% CI [-0.76; 0.021]
TssAFlnk=Flanking Active TSS, r(16)=-0.68, P=0.0019, 95% CI [-0.87; -0.31]

TxFlnk=Transcr. at gene 5’ and 3’, r(16)=-0.63, P=0.0049, 95% CI [-0.85; -0.23]
Tx=Strong transcription, r(16)=-0.26, P=0.30, 95% CI [-0.65; 0.24]
TxWk=Weak transcription, r(16)=-0.40, P=0.10, 95% CI [-0.73; 0.083]
EnhG=Genic enhancers, r(16)=-0.49, P=0.04, 95% CI [-0.78; -0.028]
Enh=enhancers, r(16)=-0.65, P=0.0034, 95% CI [-0.86; -0.26]
ZNF/Rpts=ZNF genes & repeats, r(16)=-0.28, P=0.26, 95% CI [-0.66; 0.21]
Het=Heterochromatin, r(16)=-0.43, P=0.072, 95% CI [-0.75; 0.042]
TssBiv=Bivalent/Poised TSS, r(16)=-0.31, P=0.22, 95% CI [-0.66; 0.19]
BivFlnk=Flanking Bivalent TSS/Enh, r(16)=-0.44, P=0.068, 95% CI [-0.75; 0.035]
EnhBiv=Bivalent Enhancer, r(16)=-0.53, P=0.022, 95% CI [-0.80; -0.091]
ReprPC=Repressed PolyComb, r(16)=-0.51, P=0.031, 95% CI [-0.79; -0.055]
ReprPCWk=Weak Repressed PolyComb, r(16)=-0.53, P=0.025, 95% CI [-0.80; -0.080]
Quies=Quiesent/low r(16)=-0.40, P=0.099, 95% CI [-0.73; -0.080]

Supplementary Figure 4. *AQP5* and *SLC23A1* expression and comparison of *SLC23A1* expression with DNA methylation in the SYSCOL cohort.

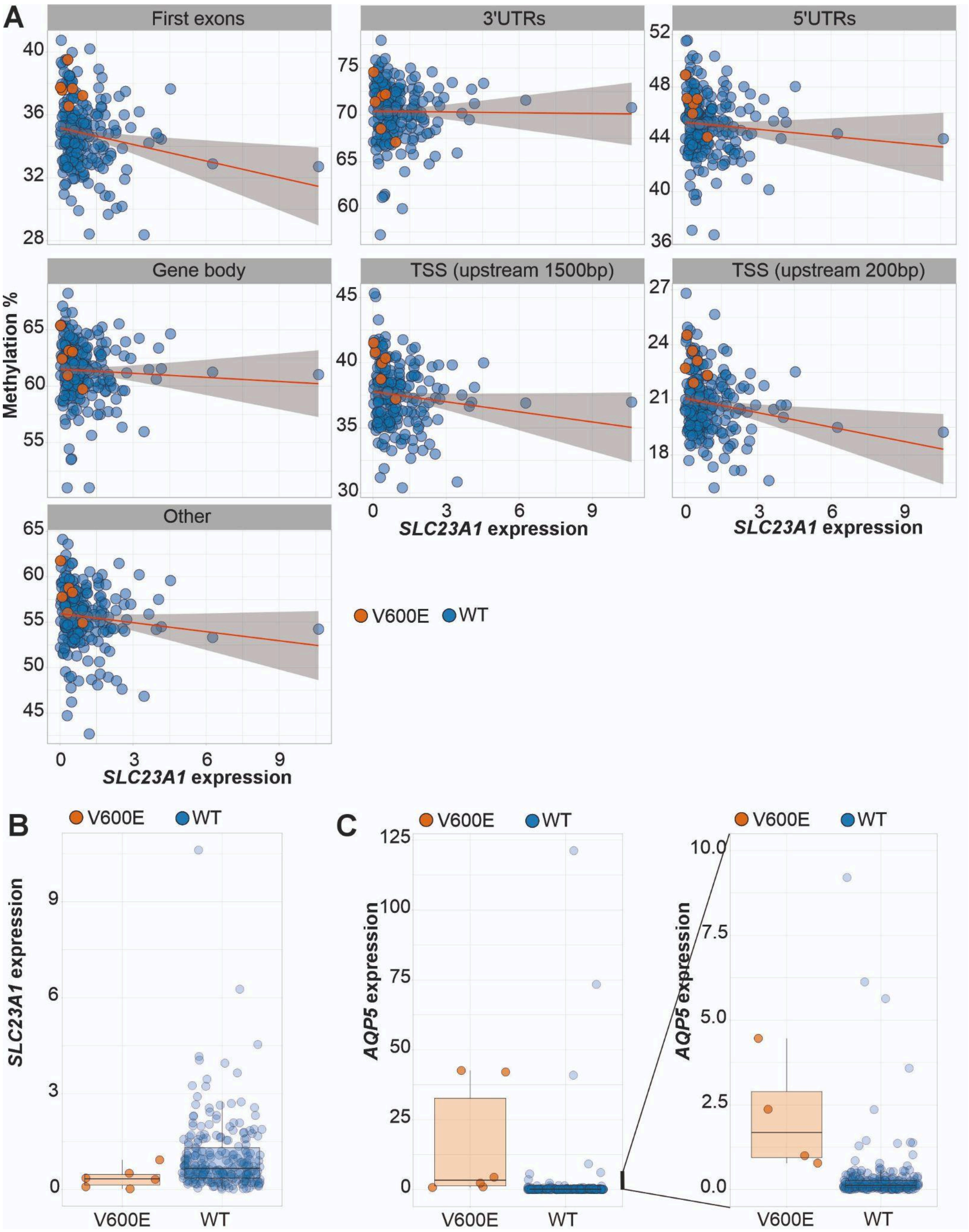

Supplementary Figure 4. SLC23A1 expression levels in a cohort of 219 MSS CRC samples with both methylation and RNA-sequencing data available. (A) SLC23A1 expression and average methylation at different genomic regions. 6 BRAF V600E and 213 WT samples had both expression and methylation data. Abbreviations and the Pearson correlation coefficients are listed below.

First exons, r(217)=-0.18, P=0.0078, 95% CI [-0.30; -0.048]

3' UTR= 3'untranslated region, r(217)=-0.011, P=0.88, 95% CI [-0.14; 0.12]
5' UTR= 5' untranslated region, r(217)=-0.088, P=0.20, 95% CI [-0.22; 0.045]
Gene body, r(217)=-0.053, P=0.44, 95% CI [-0.18; 0.080]
TSS (upstream 1500bp) = 1500bp upstream of transcription start site, r(217)=-0.12, P=0.068, 95% CI [-0.25; 0.0092]
TSS (upstream 200bp) = 200bp upstream of transcription start site, r(217)=-0.17, P=0.010, 95% CI [-0.30; -0.042]
other, r(217)=-0.11, P=0.098, 95% CI [-0.24; 0.021]
(B) SLC23A1 expression and (C) AQP5 expression in 6 BRAF V600E and 233 WT samples. The subpanel next to panel C excludes samples with extremely high AQP5 expression.

Supplementary Figure 5. Comparison of different linear model results.

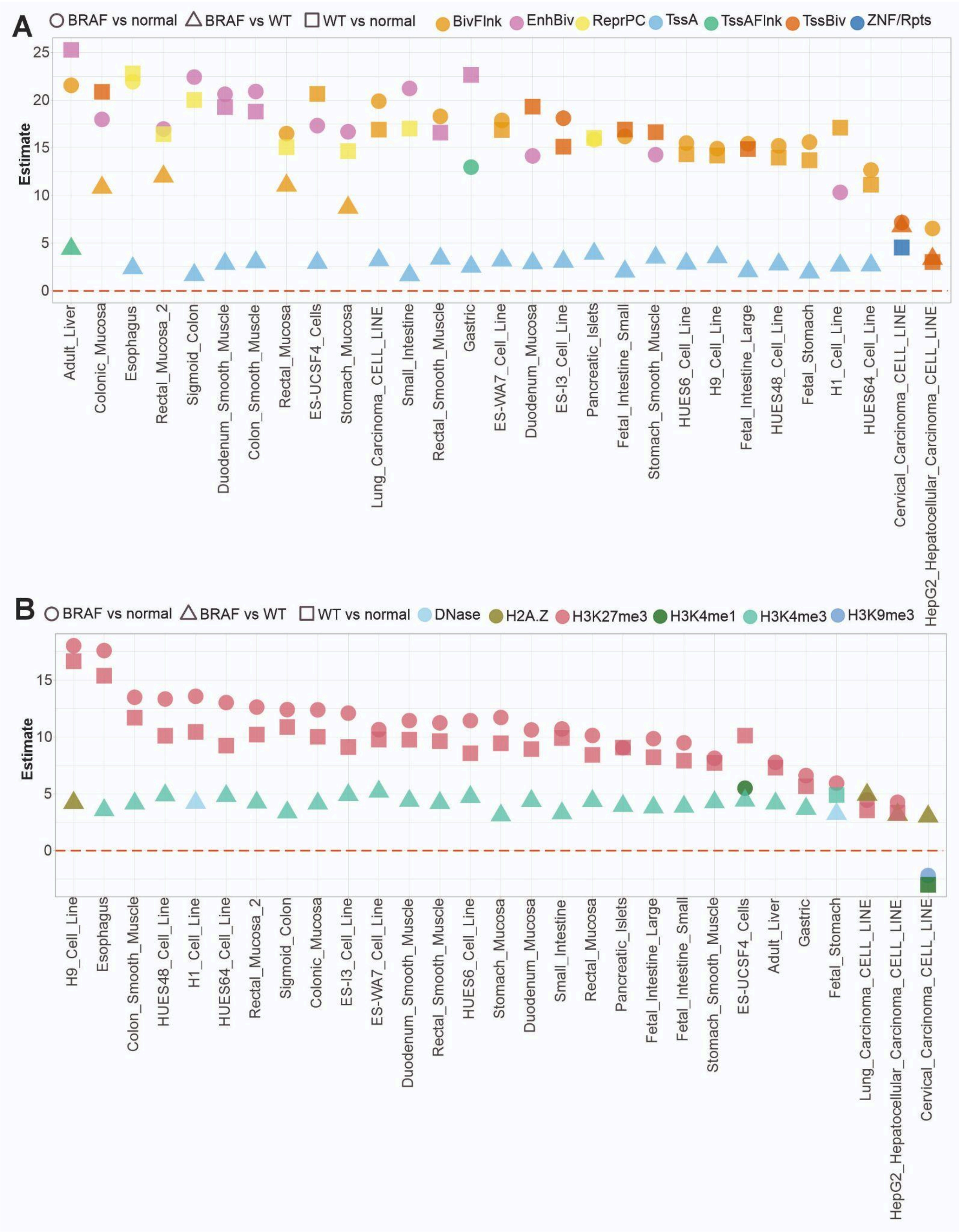

Supplementary Figure 5. The most significant linear model result for each DNA methylation analysis between colorectal tumors and normal colon (MSS BRAF V600E vs normals, MSS BRAF V600E vs MSS WT, and MSS WT vs normals) in different cell/tissue type is presented. ***(A)*** *Chromatin state and* ***(B)*** *Histone mark annotations were provided by*

Roadmap Epigenomics. The estimates are greater in tumors vs normals analyses (circles, squares) compared to moderate differences in the BRAF V600E vs WT analyses (triangles). In the tumors vs tumors analysis, the most significant result is TssA (active TSS, blue triangles in A) or H3K4me3 (light green triangles in B) for most of the cell types analyzed. For H9 ESC, lung carcinoma cell line, hepatocellular carcinoma cell line and cervical carcinoma cell line the most significant result is H2AZ. In comparisons of both WT tumors to normals and BRAF V600E tumors to normals, the mean DNA methylation level was most often elevated at chromatin with the repressive H3K27me3 mark (pink circles and squares in B) , likely reflecting methylation changes characteristic for tumorigenesis in general. TssA=Active Transcription Start Site (TSS), BivFlnk=Flanking Bivalent TSS/Enhancer, EnhBiv=Bivalent Enhancer, ReprPC=Repressed PolyComb, TssAFlnk=Flanking Active TSS, TssBiv=Bivalent/Poised TSS, ZNF/Rpts=ZNF genes & repeats.

iCAN consortium author information

Aurora Taira[1,2], Riku Katainen[1,2,3], Niko Välimäki[1,2], Ari Ristimäki[2,5], Toni T. Seppälä[2,6,7,8,9], Laura Renkonen-Sinisalo[2,6], Anna Lepistö[2,6], Selja Koskensalo[11], Kimmo Palin[1,2,9], Kristiina Rajamäki[1,2] & Lauri A. Aaltonen[1,2,9]. A full list of members and their affiliations appears in the Supplementary Information (**iCAN banner authorship list**).
